# Epigenomic analysis reveals a dynamic and context-specific macrophage enhancer landscape associated with innate immune activation and tolerance

**DOI:** 10.1186/s13059-022-02702-1

**Published:** 2022-06-24

**Authors:** Ping Zhang, Harindra E. Amarasinghe, Justin P. Whalley, Chwen Tay, Hai Fang, Gabriele Migliorini, Andrew C. Brown, Alice Allcock, Giuseppe Scozzafava, Phalguni Rath, Benjamin Davies, Julian C. Knight

**Affiliations:** 1 Chinese Academy of Medical Science Oxford Institute, University of Oxford, Oxford, UK; 2 Wellcome Centre for Human Genetics, University of Oxford, Oxford, UK; 3 Ruijin Hospital, Shanghai Jiao Tong University School of Medicine, Shanghai, China

## Abstract

**Background:**

Chromatin states and enhancers associate gene expression, cell identity and disease. Here, we systematically delineate the acute innate immune response to endotoxin in terms of human macrophage enhancer activity and contrast with endotoxin tolerance, profiling the coding and non-coding transcriptome, chromatin accessibility and epigenetic modifications.

**Results:**

We describe the spectrum of enhancers under acute and tolerance conditions and the regulatory networks between these enhancers and biological processes including gene expression, splicing regulation, transcription factor binding and enhancer RNA signatures. We demonstrate that the vast majority of differentially regulated enhancers on acute stimulation are subject to tolerance and that expression quantitative trait loci, disease-risk variants and eRNAs are enriched in these regulatory regions and related to context-specific gene expression. We find enrichment for context-specific eQTL involving endotoxin response and specific infections and delineate specific differential regions informative for GWAS variants in inflammatory bowel disease and multiple sclerosis, together with a context-specific enhancer involving a bacterial infection eQTL for *KLF4*. We show enrichment in differential enhancers for tolerance involving transcription factors NFκB-p65, STATs and IRFs and prioritize putative causal genes directly linking genetic variants and disease risk enhancers. We further delineate similarities and differences in epigenetic landscape between stem cell-derived macrophages and primary cells and characterize the context-specific enhancer activities for key innate immune response genes *KLF4*, *SLAMF1* and *IL2RA*.

**Conclusions:**

Our study demonstrates the importance of context-specific macrophage enhancers in gene regulation and utility for interpreting disease associations, providing a roadmap to link genetic variants with molecular and cellular functions.

**Supplementary Information:**

The online version contains supplementary material available at 10.1186/s13059-022-02702-1.

## Introduction

Macrophages play a critical role in immune homeostasis and tissue inflammation as well as immune clearance of bacteria, viruses and fungal pathogens [1, 2]. Dysregulation of macrophage functions is a key mechanism underlying susceptibility and pathogenesis of many autoimmune disorders, chronic inflammatory diseases, infections and cancer [3]. Upon immune stimuli such as bacterial lipopolysaccharide (LPS) exposure, macrophages can follow different functional programs. The initial acute response reprograms macrophages into a pro-inflammatory state. However, repeated immune activation such as occurs in severe infection leads to tolerance, with macrophages entering a refractory state characterized by diminished pro-inflammatory signalling upon secondary stimulation and where inappropriate or prolonged this can contribute to the pathogenesis of diseases such as sepsis [4]. These functional phenotypes are associated with substantial reorganisation of the chromatin/epigenetic architecture and binding of specific transcription factors [5–7]. Most macrophage epigenetic studies have focused on the acute response to LPS or secondary responses following LPS pre-exposure in monocytes [6–9], but acute exposure following chronic, low-dose stimulation has been relatively unexplored despite reflecting more accurately the phenotype of endotoxin tolerance. Induced pluripotent stem cell-derived macrophages (iPSMs) provide an important model system with opportunity for genetic manipulation, but it is unclear to what extent they reflect the epigenetic landscape of response seen in primary macrophages [10, 11]. Moreover, whether context-specific regulatory DNA sequences are associated with functional genetic variants and which gene networks and expression signatures are key modulators is also poorly understood. Such knowledge is important as expression quantitative trait (eQTL) mapping and genome-wide association studies (GWASs) have shown that common genetic variants are important drivers of individual differences in the immune response and disease susceptibility. These variants, typically single nucleotide polymorphisms (SNPs), are most commonly found in non-coding genomic regions and may act to modulate gene regulation in a highly tissue and context-specific manner dependent on immune stimuli and host-pathogen environment [12–15]. Improved understanding of the epigenetic landscape of immune activation and tolerance in macrophages offers the opportunity to understand such variants and the functional basis of genetic associations [16, 17].

In this study, we describe the spectrum of human macrophage enhancers under innate immune response and tolerance conditions and the regulatory networks between these enhancers and various biological processes, including gene expression, splicing regulation, transcription factor binding and enhancer RNA (eRNA) signature. We find that the vast majority of enhancers that are upregulated upon acute response subsequently undergo endotoxin tolerance and that enhancers modulated by innate immune state are significantly enriched for regulatory genetic variants and associated with gene expression levels. We demonstrate the extent of shared chromatin accessibility in primary macrophage and iPSMs relative to other immune cell types. We prioritize the disease-gene interactions and further demonstrate how the variant-containing enhancers regulate gene expression in a human macrophage model system through CRISPR interference (CRISPRi).

## Results

### Epigenetic and transcriptional changes upon LPS response and tolerance

**Fig. 1:**
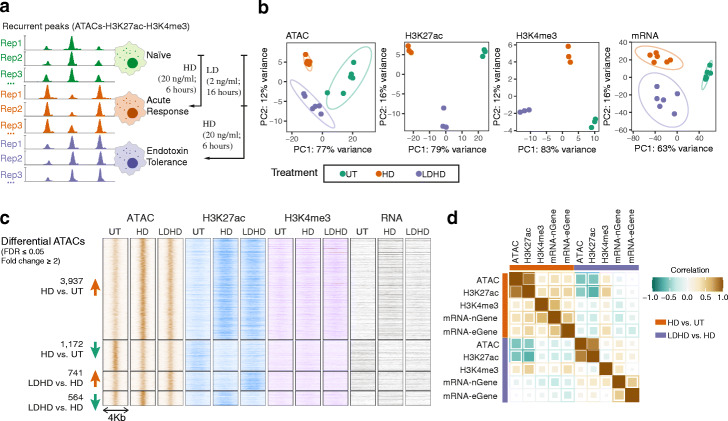
Macrophage epigenetic landscape for acute and tolerant innate immune response. **a** Schematic diagram of recurrent epigenetic marks (occurring in at least 30% samples) with varying signals in different macrophage states. **b** Principal component analysis (PCA) of the chromatin accessibility (*n* = 6), H3K27ac/ H3K4me3 activity (*n* = 3) and gene expression (*n* = 6) upon LPS treatments. Each dot represents an independent sample and colours indicate different macrophage states. **c** Heatmaps showing ATAC-seq and H3K27ac signals at differential ATAC regions (FDR < 0.05, > 2-fold changes in HD vs. UT and/or LDHD vs. HD). The normalized mean signals across sample replicates in each condition were plotted. **d** Correlations of log2 fold change of ATACs, H3K27ac, H3K4me3 and gene expression (nGene: nearest gene to ATAC peaks; eGene, genes linked with ATAC peaks based on MDM eQTLs) upon LPS response (HD vs. UT) and LPS tolerance (LDHD vs. HD). See also Fig. S1

We first sought to generate epigenetic maps of innate immune activation and tolerance in primary human macrophages differentiated from circulating blood monocytes (monocyte-derived macrophages; MDMs). We compared three innate immune states: naïve unstimulated MDMs, MDMs exposed to high-dose LPS (HD) (acute response) or MDMs exposed to low-dose LPS and subsequent high-dose LPS challenge (LDHD) (endotoxin tolerance) [4]. We assayed chromatin accessibility (ATAC-seq) and two informative histone modifications, H3K27ac (mark for active enhancers) and H3K4me3 (promoters) together with total RNA sequencing to profile coding and non-coding RNA (Fig. 1a). Principal component analysis showed the naïve MDMs clustered together and were clearly distinct from acute response and tolerance states for all these chromatin profiles as well as gene expression (Fig. 1b). In total, 8.5% (5985 out of 70,100) of accessible chromatin peaks present in at least 30% of samples (denoted recurrent ATAC peaks; ATACs), 20.3% (5729 out of 28249) of H3K27ac and 3.3% (656 out of 20103) of H3K4me3 peaks were found to be differential, changing during HD vs. untreated (UT) and/or LDHD vs. HD (FDR < 0.05; fold change > 2; Fig. 1c and S1a).

The vast majority of the differential ATACs and/or H3K27ac peaks associated with the acute response were found to be subject to tolerance (Fig. 1c). We determined that 54.6% (308 out of the 564) of differential ATACs and 70.2% (1019 out of 1451) differential H3K27ac peaks downregulated in LDHD vs. HD were upregulated in the HD vs. UT condition (Additional file 1: Fig. S1b). These differential ATACs and H3K27ac peaks were mainly located in intronic and distal intergenic regions (> 60%; Additional file 1: Fig. S1a). Overall, 31.5% (1887 out of 5985) of differential ATACs overlap H3K27ac peaks and displayed clear correlation upon responses (Fig. 1d), indicating a substantial fraction of these accessible ATACs are likely enhancers.

### Coincident relationship with genetic drivers of individual differences in gene expression

**Fig. 2:**
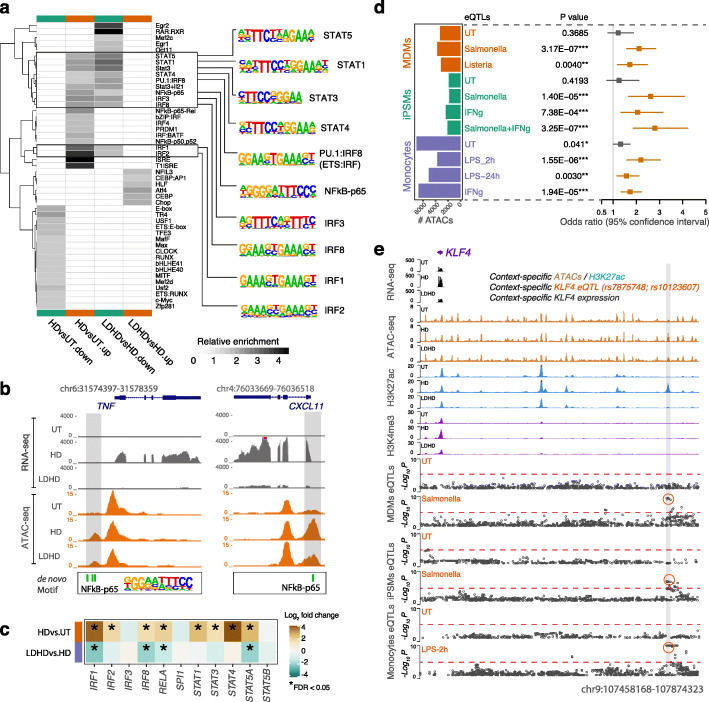
Transcription factor motifs, context-specific eQTLs and differential chromatin accessibility and modifications. **a** Heatmap showing the enrichment of transcription factor (TF) binding motifs in differential ATAC versus non-differential ATAC regions. Greyscale values indicate enrichment levels. Only statistically significant results (FDR < 0.05) are shown. The LPS treatment conditions are shown on the *x*-axis and enriched TF motif clusters on the *y*-axis. Motifs enriched in both upregulated ATAC regions upon LPS response and downregulated ATAC regions upon tolerance are highlighted on the right panel. **b** Example of proinflammatory cytokine and chemokine genes *TNF* (left panel) and *CXCL11* (right panel), for which the promoter regions have differential ATAC regions containing NFkB motifs. The average sequencing depth (normalized per million mapped reads) for RNA-seq and ATAC-seq from 6 independent biological replicates are shown on the y axis. **c** Heatmap showing the log2 fold change of gene expression (upper: HD vs. UT; lower: LDHD vs. HD) for the enriched TFs. Gene upregulation is indicated in brown and downregulation in green. **d** Forest plot showing enrichment of differentially expressed genes linking differential ATACs compared to non-differential ATACs using MDM eQTLs (upper), iPSM eQTLs (middle) or monocytes eQTLs (lower). The odds ratio and *p*-values are depicted and were calculated using a two-tailed Fisher test. In case multiple genes were attributed to an ATAC peak, the gene associated with the most significant eQTL that overlaps the peak was selected for the analysis. **e** *KLF4* locus showing differential chromatin accessibility and H3K27ac (region highlighted in grey) harbouring context-specific *KLF4* eQTLs (red circles). See also Fig. S2

Transcription factors (TFs) bind to specific DNA consensus sequences and are master regulators of gene expression and cell identity. We reasoned that identification of context-specific ATAC regions and linked genes would enable the systematic discovery of functional regions for which regulatory patterns were likely to be driven by specific TFs. We identified 47 out of 428 known TF sequence recognition motifs were enriched in differential ATAC regions (FDR < 0.05 and fold change > 1.5; Fig. 2a). Of these, 10 motifs were linked to the tolerized phenotype, i.e. highly enriched in ATAC regions that were upregulated upon LPS but downregulated in response to LPS re-exposure (Fig. 2a right panel). These TFs, including genes encoding signal transducer and activator of transcription factors (STATs), nuclear factor-κB (NF-κB) and interferon regulatory factors (IRFs), are known to be involved in tolerized pathways. For example, two tolerance-associated ATAC regions (highlighted in Fig. 2b) harbouring the NFkB-p65 motif aligned consistently with the dynamics of gene expression for two known NFkB-p65 target genes *TNF* and *CXCL11*. Genome-wide, we found that the majority of TFs with binding motifs enriched in tolerance-associated regions also exhibited consistent gene expression patterns showing endotoxin tolerance (Fig. 2c). Together, these observations suggest that the differential ATACs are likely to be functionally dependent on their associated TFs, providing putative connections between TFs and regulatory functions.

The effect of eQTLs on gene expression varies across tissues and contexts, and they are highly enriched in cis-regulatory elements [13, 18]. We hypothesized that functional context-specific and disease-relevant eQTLs are more likely be enriched in context-specific enhancer regions. Consistent with previous observations, we found cis-eQTLs identified in MDMs [14] are more enriched in macrophage accessible chromatin regions (recurrent ATAC peaks) compared with all common variants (Additional file 1: Fig. S2a; ~ 8% per eQTL dataset vs. 3% of all common dbSNPs). We observed similar enrichment using primary monocytes [12], iPSMs [19] and sepsis eQTLs [20] (Additional file 1: Fig. S2a), suggesting a shared effect in similar tissues and infectious environment. Interestingly, we found greater proportions of context-specific eQTLs (eQTLs that are associated with gene expression upon immune stimulations but not in naïve state) overlapped the corresponding differential ATACs (Additional file 1: Fig. S2b). For example, 328 out of 3729 (8.8%) eQTLs that were only identified in Salmonella treated MDMs [14] reside in differential ATACs compared to 190 out of 3297 (5.8%) of naïve-specific eQTLs (OR = 1.6; *p* = 1.2e−06). We also reasoned that the genes linked with differential ATACs through context-specific eQTLs would be differentially expressed. Indeed, we observed significant enrichment of differentially expressed genes linking differential ATACs compared to non-differential ATACs, using eQTLs only identified in stimulated macrophages or monocytes but not eQTLs that were only identified in the naïve state (Fig. 2d). These genes included key components involved in TLR4 inflammatory responses to infection, such as *KLF4* (Additional file 2: Table S1; Fig. 2e), for which the context-specific eQTLs link differential enhancer activity and/or chromatin accessibility that were positively correlated with mRNA levels upon LPS treatments.

### eRNA signatures in different macrophage states

**Fig. 3:**
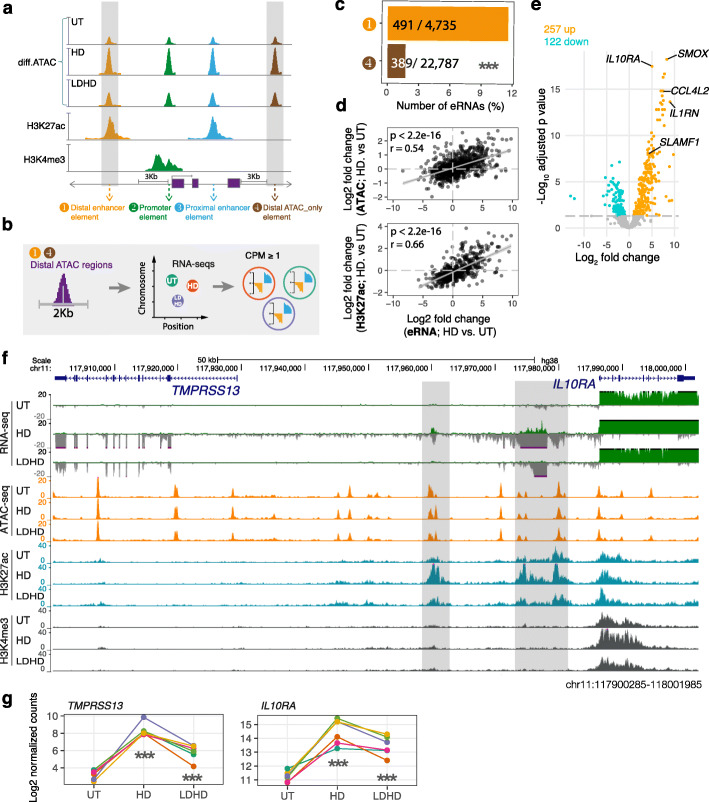
Enhancer RNAs and differential epigenetic macrophage landscape. **a** Schematic defining distal regulatory DNAs by epigenetic signatures and proximity to expressed gene (maximum TPM ≥ 1 across samples) bodies in MDMs. **b** Outline of context-specific eRNA analysis procedure. **c** A bar plot showing the percentage of distal enhancers (orange) and distal ATAC-only regions (brown) that contain expressed eRNAs (CPM ≥1). *p* value was calculated by two-tailed fisher test. ***p < 0.001. **d** Correlation of log2 fold change of eRNAs and ATAC regions (upper) or H3K27ac peaks (lower) in HD vs. UT. Pearson’s r and *p* values are shown. **e** Volcano plot showing differentially expressed eRNAs following acute LPS stimulation. The nearest gene for the top three hits are highlighted. **f** Example of differential eRNAs proximal to differentially expressed genes (*TMPRSS13* and *IL10RA*) for acute LPS response and tolerance. The average sequencing depth for RNA and epigenetic marks are shown on the y axis. Regions with differential eRNAs, ATACs and H3K27ac are highlighted in grey. **g** mRNA expression of each gene indicated in **f** was compared upon LPS response (HD vs. UT) and LPS tolerance (LDHD vs. HD) with samples from 6 different donors (indicated by different colour dots with linked lines). *p* value was calculated by linear regression. ***p < 0.001

Enhancer activity is associated with recruitment of RNA polymerase II and transcription of non-polyadenylated, bidirectional enhancer RNAs (eRNAs) [21]. As described above, we observed a clear correlation of differential ATAC regions with enhancer activities marked by H3K27ac. To further determine whether the differential ATAC signals were concordant with putative enhancer activities associated with eRNAs, we localized the eRNAs using ATAC-seq and total RNA-seq in MDMs and assessed (i) whether the eRNAs were regulated by LPS treatments and (ii) whether eRNAs modulated by LPS are positively related to ATACs and H3K27ac profiles. We focused our analysis on distal ATAC regions that did not overlap with known expressed transcripts in MDMs (± 3000 bp from both transcription start site and end site) (Fig. 3a, b). We found the distal H3K27ac-containing ATAC regions were more likely to have highly expressed eRNAs relative to distal ATAC only regions (Fig. 3c), indicating H3K27ac modification plays a major role in controlling eRNA expression. Furthermore, the eRNA signature was positively correlated with both ATAC and H3K27ac activity (Fig. 3d). In total, 379 out of 880 detectable eRNAs (CPM ≥ 1) were differentially expressed upon LPS (Fig. 3e; Additional file 2: Table S2). These differential eRNAs are proximal to key immune modulators that showed concordant LPS-induced expression patterns (Fig. 3f, g). Such non-coding regions are likely to be highly functional, providing targets for experimental manipulations to investigate connections between regulatory DNAs/RNAs and genes

### Annotating genome-wide association signals and therapeutic implications using differential ATACs

**Fig. 4:**
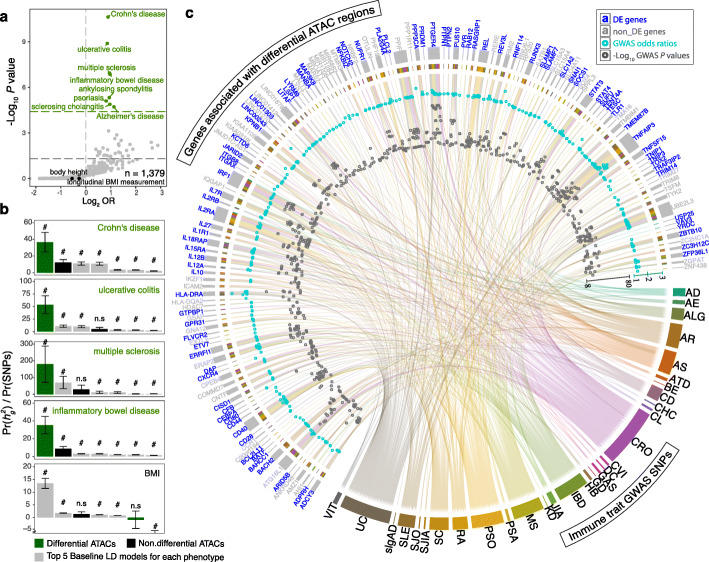
GWAS enrichment and macrophage chromatin state. **a** Enrichment of GWAS risk SNPs that are located in differential ATACs amongst each trait relative to other traits (1,379 EFO term). The horizontal green dash line represents the Bonferroni-adjusted *p* value of 0.05. **b** Bar plots showing the enrichment of the annotations of macrophage differential ATACs (green), non-differential ATACs (black) and the top5 (enrichment *p* values) baseline LD score models (grey) in GWAS signals for Crohn’s disease, ulcerative colitis, inflammatory bowel disease traits, multiple sclerosis or BMI. Bars are ordered by enrichment magnitude in each trait. Error bars represent jackknife standard errors around the estimates of enrichment as described in [24]. #: FDR < 0.05 after correction for all the annotations tested. n.s: not significant. **c** Chord Diagram of GWAS SNPs (either leads or proxies) in differential ATAC regions that associate with differential risk to immune disorders. ATACs were assigned to expressed genes through their genomic locations (nearest RefSeq genes). The connecting lines indicate the lead SNPs for each GWAS association of different studies. The cyan and grey dots indicate the median odds ratios and median –log10 *p* values of the associations, respectively. Colours in circle above cyan dots represent the linked immune traits. The differentially expressed genes in HD vs. UT and/or LDHD vs. HD are highlighted in blue. AD, autoimmune disease; AE, atopic eczema; ALG, allergy; AR, allergic rhinitis; AS, ankylosing spondylitis; ATD, autoimmune thyroid disease; BE, Barrett’s esophagus; CD, celiac disease; CHC, chronic hepatitis C virus infection; CL, cholelithiasis; CRO, Crohn’s disease; CVI, common variable immunodeficiency; DC, dental caries; GAS, Gallstones; GD, Graves’ disease; HB, hepatitis B virus infection; IBD, inflammatory bowel disease; JIA, juvenile idiopathic arthritis; KD, mucocutaneous lymph node syndrome; MS, multiple sclerosis; PSA, psoriatic arthritis; PSO, psoriasis; RA, rheumatoid arthritis; SC, sclerosing cholangitis; SJIA, systemic juvenile idiopathic arthritis; SJO, Sjogren’s syndrome; SLE, systemic lupus erythematosus; slgAD, selective IgA deficiency disease; UC, ulcerative colitis; VIT, vitiligo*.* See also Fig. S3

**Fig. 5:**
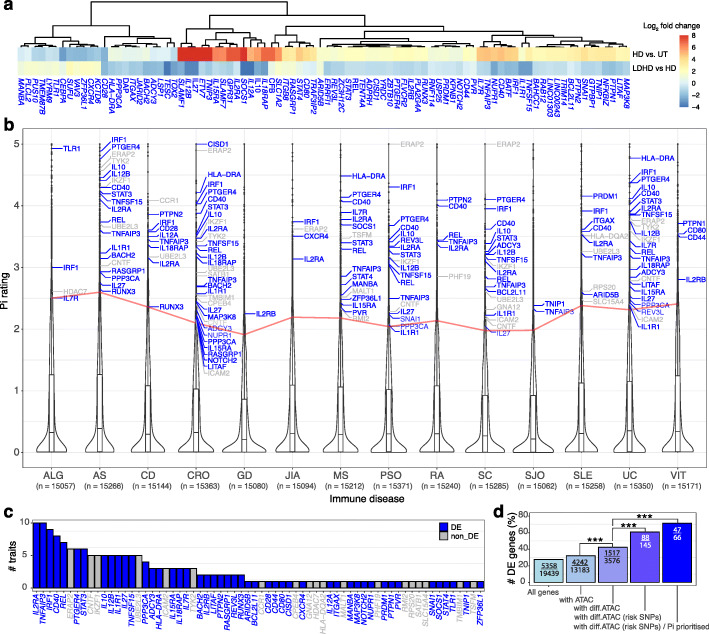
Drug target prioritization (priority index, Pi) for immune disease risk genes in macrophages. **a** Heatmap showing the log2 fold change upon HD vs. UT or LDHD vs. HD of differentially expressed immune disease risk genes in MDMs (as highlighted in Fig.4c). **b** Violin plot showing the distribution of the Pi rating scores of all genes for which a Pi rating was computed. The number of genes analysed for each trait is indicated in a parenthesis. The Pi prioritized risk genes identified by macrophage chromatin states and with Pi rating scores more than the 95th percentile (the horizontal red line) are listed (DE gene in blue). **c** Bar plot showing the number of traits associated with each Pi prioritized risk gene. **d** Bar plot of the percentage of DE genes in each group as indicated on the *x*-axis. Numbers of genes (number of DE genes/total number of genes) in each group are indicated within the bars. *p* values were calculated by two-tailed Fisher test (***p < 0.001). ‘All genes’: all MDMs expressed genes; ‘with ATAC’: genes with proximal recurrent ATACs; ‘with diff.ATAC’: genes with proximal differential ATACs; ‘with diff.ATAC (risk SNPs)’: genes with risk-SNP-containing differential ATACs; ‘with diff.ATAC (risk SNPs)/Pi prioritized’: Pi highly rated genes with diff.ATAC (risk SNPs). See also Fig. S4

Inflammation and the immune response are critical pathological processes underlying many common diseases. Thousands of genomic risk loci for these diseases, most of which are non-coding, have been identified though GWASs [22, 23]. We found significant enrichment of SNPs associated with immune-related disorders, such as Crohn's disease (CRO), ulcerative colitis (UC) and multiple sclerosis (MS), in differential ATAC regions relative to non-differential ATAC regions (Fig. 4a). For example, 14% (155 out of 1081) GWAS SNPs (either lead or proxy SNPs with *r*^2^ > 0.8) associated with Crohn’s disease resided in differential ATAC regions, relative to 8% (81,235 out of 982,296) of other traits. (*P* = 3.3e−11, OR = 1.9; Fig. 4a). Similar results were observed when we restricted the analysis using the GWAS lead SNPs only (Additional file 1: Fig. S3a). On average, about 4% of the independent risk loci have overlap with differential ATAC peaks across the top enriched disease traits (Additional file 1: Fig. S3b). We next quantified the enrichment in GWAS signals of the macrophage differential ATAC regions using stratified LD Score Regression [24] (see Methods). We found strong enrichments (Pr(*(*${h}_g^2$) / Pr(SNPs)) of the differential ATAC regions in GWAS signals for CRO, US, MS and IBD (>70X on average, Fig. 4b; Additional file 2: Table S3), which were much weaker or not significant with the non-differential ATACs. It is important to note that these significant enrichments only occur in immune-relevant traits, but not in other diseases and quantitative traits such as body mass index and height measurements (Fig. 4a, b; Additional file 2: Table S3). In total, there were 416 unique immune disease associated GWAS risk SNPs in 194 differential ATAC peaks proximal to 145 genes, of which 61% (88 out of 145) were differentially expressed upon LPS treatments (Fig. 4c and Fig. 5a; Additional file 2: Table S4), including known risk genes for top enriched disease traits (CD, UC and MS) [25, 26] (for instance, *TNFSF15*, *STAT3 and HLA-DRA*), known immune regulators (for instance, *IL12A* in celiac disease and autoimmune disease, and *IRF1* in CD, allergy and autoimmune diseases), as well as yet uncharacterized non-coding RNA genes (Additional file 1: Fig. S3c-d).

We have recently established a genetics-led drug target prioritization approach (Priority index; Pi) to identify potential therapeutic targets in immune traits using immune disease GWAS, functional immunogenomics and gene interaction network [27]. To access the therapeutic implications of the macrophage enhancers, we intersected the Pi resource [28] with the disease-gene interactions identified in macrophages. We retrieved the Pi ratings for 70.6% (327 out of 463) of the interactions we had identified (Fig. 5b). This involved 14 immune traits, and we found that interaction-associated Pi ratings are higher than those of the other genes in a given trait (Additional file 1: Fig. S4; Additional file 2: Table S5). In total, 176 (53.8%) out of the 327 disease-gene pairs involving 66 unique genes have high ratings (> 95th percentile; Fig.5b), for which 71% of the genes are differentially expressed upon LPS treatments (Fig. 5b, c highlighted in blue), including the top ranked genes IL2RA and TNFAIP3 that were highly rated in 10 different traits (Fig. 5c). We hypothesized that the variant-containing differential enhancers would be more likely functional and hence associate with differential gene expression. Indeed, we determined that the differentially expressed (DE) genes were significantly enriched in risk genes relative to those genes without risk SNPs (OR = 2.2; *P* = 6.9e−06; Fig. 5d, 4th bar vs. 3rd bar). The enrichment was further enhanced when we analysed those genes highly rated by Pi (OR = 3.4; *P* = 2.1e−06; Fig. 5d, 5th bar vs. 3rd bar).

### Shared chromatin accessibility in primary macrophage and iPSMs upon LPS response and tolerance

**Fig. 6:**
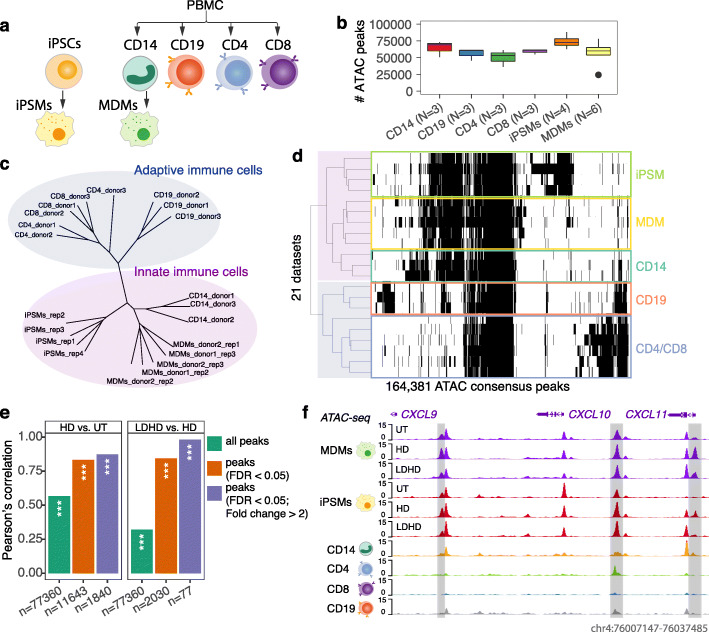
Differential chromatin accessibility across cell types and informativeness of context-specificity. **a** Purified human innate and adaptive cell types/models analysed for chromatin accessibility by ATAC-seq profiling. **b** Bar plot showing the median number of recurrent ATAC peaks on the *y*-axis and sample size on the *x*-axis for each cell type. One outlier (black dot) was excluded from the clustering analysis in **c**. **c** Phylogenetic tree showing the relatedness of chromatin accessibility amongst cell types based on hierarchical clustering using Average method and Jaccard similarity measure. **d** Clustered binary matrix showing the 164,381 ATAC consensus peaks across 21 datasets derived from 5 cell types. Dark and white indicate presence and absence of recurring ATAC regions in each dataset respectively. **e** Bar plots showing correlation of log2 fold change of ATAC peaks upon HD vs. UT (left) and LDHD vs HD (right), between iPSMs and MDMs. Pearson’s *r* are shown on the *y*-axis. ***p < 0.001. **f** Example of a locus with clustered pro-inflammatory genes that have nearby tissue- (MDMs in purple, iPSMs in red, CD14 monocytes in orange, CD4 T cells in green, CD8 T cells in blue and CD19 B cells in grey) and context-specific (UT/HD/LDHD for MDMs and iPSMs) ATAC regions. The normalized signals across the replicates of each cell type are shown on the y axis. See also Figs. S5 and S6

Previous studies have revealed that closely shared signatures of regulatory genomic regions across cell types exhibit common biological functions such as enhancer activity and TFs binding [29–31]. To explore the differences and similarities of chromatin accessibility between immune cell types, we generated 21 uniformly processed ATAC-seq datasets in primary CD4/CD8 T cells, CD19 B cells, CD14 monocytes and MDMs as well as iPSMs (Fig. 6a). We identified an average of ~ 70,000 high-confidence recurrent ATAC regions per cell type derived from different healthy donors and replicates (Fig. 6b), and a total of 164,381 distinct ATAC consensus peaks, of which 55,373 were specific to a single cell type, 79,645 were active in 2 or more cell types, and 29,369 (18%) were detected in all cell types (Fig. 6d; Additional file 2: Table S6). Interestingly, unsupervised hierarchical cluster analysis showed two major clusters that clearly distinguish the adaptive and innate immune cell types, i.e. cells of the myelomonocyte lineage were clustered together compared to T cells and B cells (Fig. 6c, d).

iPSMs are reported to share striking similarities, but also show differences with primary human macrophages in terms of phenotypic, secretome and transcriptome profiles [11, 32–34]. Consistent with these previous studies, we observed significant overlap and strong correlation in genes that were differentially expressed (DE) and had differential exon usage (DEU) (Additional file 1: Fig. S5) and in differential ATAC profiles upon both LPS response and tolerance between MDMs and iPSMs (Fig. 6e). Although the LPS responses in these two macrophage models are broadly conserved, we also observed clear differences in numbers of DE genes/DEUs identified (Fig.S5), which is consistent with previous studies [32–34]*.* To directly compare the epigenetic changes upon stimulations, we counted the iPSMs ATAC-seq reads by using the recurrent peak regions identified in MDMs. As expected, the main variance of chromatin accessibility between the two cell types was explained by the cell origin (Additional file 1: Fig. S6a). When we corrected variance due to the differing cell types and donors, samples from the two cellular models tend to be clustered together based on the treatment conditions (Additional file 1: Fig. S6b). In total, we determined 56.7% (2898 out of 5019 upon LPS response) and 25.4% (331 out of 1305 upon LPS tolerance) of the differential ATACs identified in MDMs were also differential in the same direction in iPSMs (Additional file 1: Fig. S6c-d; Additional file 2: Table S7). For example, iPSMs and MDMs displayed multiple cell-type-specific (adaptive cells vs. innate cells) and context-specific ATACs in a locus containing pro-inflammatory chemokines *CXCL9/10/11* (Fig. 6f highlighted in grey). Together, these findings support the fidelity of iPSMs, relative to primary MDMs, in functions of macrophages in innate immunity, providing a useful tool for genotype-specific functional disease modelling.

### Observed context-specific gene expression is dependent on coincident enhancer activity

To explore how knowledge of the context-specific enhancers could help prioritize experimental studies to characterize causal genetic variants, we tested the effects of variant-containing enhancers on gene expression using CRISPRi. We infected iPSMs, constitutively expressing dCas9-KRAB, with lentiviral particles expressing CRISPRi single-guide RNAs (sgRNAs) that target the enhancers, and examined the effect on gene expression upon different LPS stimulations (Additional file 1: Fig. S7a). By targeting a distal enhancer containing context-specific eQTLs associated with *KLF4* (~ 337 Kb to gene TSS; Fig. 2e and Additional file 1: Fig. S7b), we observed clear reduction of *KLF4* expression upon acute LPS response (2.3-fold, *P* = 7.7e−04; Additional file 1: Fig. S7e) but not in untreated and LPS-tolerized conditions. Consistently, amongst all genes that lie approximately 2 Mb on either side of the enhancer, *KLF4* is the only one that was significantly upregulated upon LPS response and downregulated upon LPS tolerance (Additional file 1: Fig. S7c-d).

**Fig. 7:**
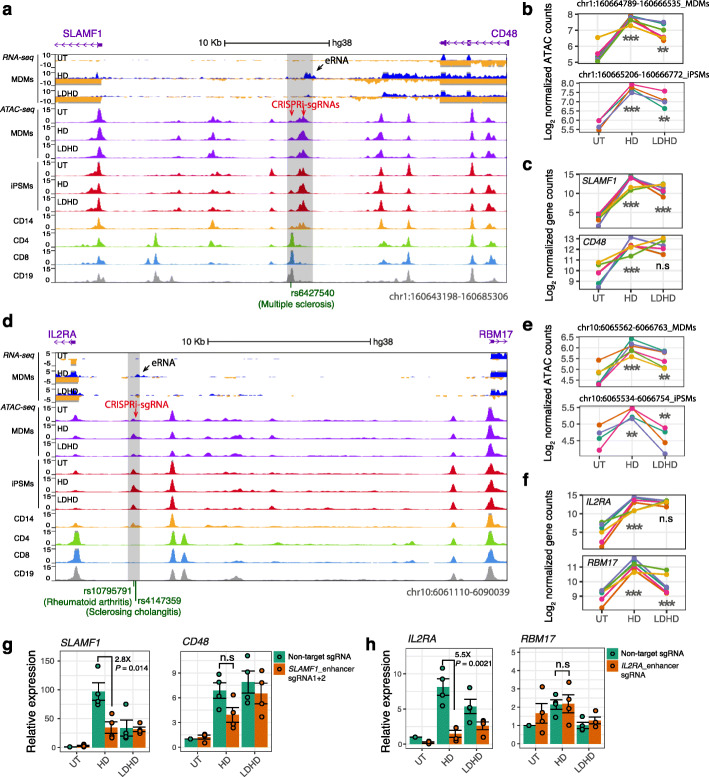
CRISPR interference-based enhancer inactivation in iPSMs. **a** A differential eRNA and ATAC region at chr1q23.3 containing a GWAS lead SNP associated with MS risk (rs6427540 [35]). The differential ATAC peak identified in both MDMs and iPSMs is highlighted in a grey bar, eRNA expression in MDMs is indicated by a black arrow, and the sgRNA sites for CRISPRi are highlighted by red arrows. The normalized RNA and ATAC signals across the replicates of each cell type are shown on the y axis. **b**, **c** Quantifications of the ATAC peak (highlighted in **a** in MDMs (**b**, upper panel) and iPSMs (**b**, lower panel) and the expression of its proximal genes *SLAMF1* (**c**, upper panel) and *CD48* (**c**, upper panel) in MDMs. Each coloured line represents samples from one donor across different treatment conditions. The significance was determined by linear regression, comparing normalized sequencing counts upon LPS response (HD vs. UT) or LPS tolerance (LDHD vs. HD). ***p < 0.001; **p < 0.005; n.s: not significant. **d**–**f** A MDMs/iPSMs shared differential ATAC peak at chr10p15.1 contains GWAS lead SNPs associated with RA (rs10795791 [36]) and SC disease risk (rs4147359 [37]) (**d**) and the quantifications of the ATAC (**e**) and proximal gene expression (**f**) as described above. **g**, **h** Bar plots of gene expression, measured using qRT-PCR normalized to *GAPDH* (2^–∆∆Ct^) in CRISPRi edited iPSMs with either a non-target sgRNA control (green bars) or sgRNAs targeting enhancers as indicated (red bars). Error bars represent SEM of 4 independent replicates. *P* value was calculated by two-tailed Student’s *t*-test. See also Figs. S7, S8 and S9

We next examined two distal enhancers containing disease risk SNPs. A single MS GWAS lead SNP rs6427540 [35], which was located in a differential ATAC peak between the transmembrane receptor genes *SLAMF1* (also called CD150; ~ 17 Kb to gene TSS) and *CD48* (~ 20 Kb to TSS), overlapped a clear eRNA that was only observed upon acute response (Fig. 7a). We observed coincident dynamics of chromatin accessibility and gene expression upon immune stimulations at this locus (Fig. 7b, c). As predicted from the above-described associations, we found significantly lower *SLAMF1* expression in macrophages with enhancer-targeting sgRNAs compared with a non-targeting control sgRNA upon acute immune response (2.8-fold, *P* = 0.014; Fig. 7g left panel), but not in untreated and LPS-tolerized conditions. The enhancer activity on LPS-mediated SLAMF1 induction was also observed in terms of protein levels via flow cytometry (Additional file 1: Fig. S8). We found no significant differences for the other nearby gene, *CD48* (Fig. 7g right panel; Additional file 1: Fig. S8). Two lead SNPs (rs10795791 [36] in RA and rs4147359 [37] in SC) were located within the differential enhancer that is proximal to the top Pi prioritized gene *IL2RA* (~ 4 Kb to TSS), and *RBM17* (~ 22 Kb) (Fig. 7d–f). Similarly, we observed 5.5-fold reduction of IL2RA expression by targeting this enhancer (*P* = 0.0021; Fig. 7h). It is also important to note that the expressions of the target genes were not affected by targeting the non-differential enhancers in the same locus (Additional file 1: Fig. S9). Together, these observations suggest that the transcriptional regulation of prioritized target genes may be primed by cell-type- and stimulation-specific enhancer activities to influence disease risk.

## Discussion

We characterized the innate immune epigenetic and transcriptional landscape of human macrophages, acutely and in a tolerant state. We chose to focus on macrophages because they play a critical and non-redundant role in sensing the presence of infection through microbial-specific molecules and as key mediators of the innate response controlling infection. This analysis enabled us to identify a subset of regulatory enhancers and demonstrate that these differential epigenetic changes facilitate a dissection of the relationships between enhancer accessibility, TF binding, mRNA/eRNA expression and the molecular architecture of disease-associated genetic variants in human macrophages.

Genome-wide association studies have identified thousands of generic variants associated with common diseases and quantitative traits, and these variants are highly enriched in non-coding regulatory elements [17, 23, 29]. These regulatory elements are often cell type specific, regulating gene expression in different cellular states [38, 39]. We show that such genetic variants are highly enriched in stimulus-specific regulatory elements relative to steady-state regions marked by chromatin accessibility. We identified hundreds of variant-containing context-specific ATAC regions in macrophages, linking known risk genes, such as *IL2RA*, *SLAMF1*, *TNFSF15*, *STAT3*, *HLA-DRA*, *IL12A*, and *IRF1*, that help inform the functional basis of the reported diseases associations. Despite the fact that disease-associated variants are highly enriched in regulatory DNAs, a relatively small fraction of these loci can be explained by known eQTLs, even those identified in relevant cell types [40–42]. One possible explanation is that the effects of genetic variants on gene expression are strongly context-specific, relying on the right epigenetic environment to occur. Indeed, multiple reports have highlighted that the activity induced by immune stimulation conditions the effect of regulatory variants on gene expression [12, 14, 19, 20, 43]. Our results demonstrate that these context-specific eQTLs are highly enriched in context-specific enhancers that were positively correlated with target gene expression, providing evidence that overlap of generic variants with stimulus-specific enhancers for prioritizing causal disease genes are likely to be more informative. For example, we show evidence for a context-specific enhancer linking *KLF4* through eQTLs that is only present on bacterial infection in macrophages. *KLF4* encodes a transcription factor that controls the expression of anti-inflammatory genes in macrophages [44]. More relevant to infection, *KLF4* downregulation is reported in a mouse model of sepsis [45], which is consistent with the epigenetic evidence based on our data, whereby the acute LPS-stimulated enhancer is remarkably suppressed upon LPS tolerance, a hallmark of sepsis.

Context specificity for gene expression can also be achieved by differential binding of signal-dependent transcription factors (SDTFs) and lineage-determining transcription factors (pioneer TFs, e.g. PU1 in macrophages) [46, 47]. SDTFs such as LPS-induced NF-κB, STAT or IRF protein family have been reported to trigger stimulus-specific regulatory programming in open chromatin primed by PU1 [7, 46]. Our TF analysis reveals the enrichment of binding motifs for these SDTFs in differential ATAC regions upon LPS stimulation. Most of the TFs whose binding motifs were enriched in both LPS-induced and tolerized ATAC regions exhibited positively correlated gene expression patterns. Our data suggest that the functional identity of differing macrophage states induced by the LPS response and in tolerance may be controlled by the sequential PU1 and SDTF bindings, which subsequently modulate the epigenetic landscape through recruiting histone-modifying enzymes to alter chromatin accessibility, histone modifications and gene transcription. However, further investigation is needed to underpin this observation and elucidate its molecular mechanisms.

The biological functions of eRNA are associated with TF recruitment, chromatin conformation and histone modifications [48–51]. We identified novel strong eRNAs that are proximal to important immune modulators such as the *TMPRSS13/IL10RA* and *SLAMF1/CD48* regions. We demonstrate that H3K27ac modification plays a major role in controlling eRNA expression. However, based on total RNA-seq, we were able to only capture less than 1000 detectable eRNAs across conditions, and most of them are of low abundance and variable across replicates, thus reducing our ability to detect highly confident context-specific eRNAs.

Although iPSMs and MDMs exhibited broadly conserved transcriptome and epigenetic profiles in response to LPS stimulations, we observed clear differences in numbers of DE genes, DEUs and differential enhancers between the two macrophage models, which likely reflects iPSMs representing primitive, tissue resident macrophages which are known to be developmentally and functionally distinct from MDMs [52, 53]. We determined that about 50% of the differential ATACs identified in MDMs showed consistent profiles in iPSMs. Using a CRISPRi system in iPSMs, we experimentally validated the effects of three variant-containing enhancers on the expression of *KLF4* and two prioritized disease risk genes *IL2RA* and *SLAMF1* encoding cell-surface receptors and for which an approved or investigational drug was available [54]. This system provides us a valuable alternative model for future study aimed at systematically characterizing the causal interactions between stimulus-specific regulatory DNAs and associated genes and pathways and prioritising potential drug targets for disease intervention.

## Methods

### PBMCs purification and primary immune cell culture

Peripheral blood samples were obtained from healthy volunteers and PBMC isolated by Ficoll-Paque centrifugation. Monocytes (CD14+), T cells (CD4+ and CD8+) and B cells (CD19+) were separated from peripheral blood mononuclear cells (PBMCs) by positive selection with magnetic MicroBeads (Miltenyi Biotec). The isolated CD14 monocytes were differentiated into macrophages (MDMs) by culturing cells for 6 days in RPMI-1640 (Sigma) supplemented with 20% FBS (Sigma), L-glutamine (Sigma), sodium pyruvate (Sigma), non-essential amino acids (Sigma) and 20 ng/mL M-CSF (Gibco).

### Macrophage differentiation from human iPSCs

Induced pluripotent stem cell (iPSC) line (SFC841-03-01) was reprogramed from dermal fibroblasts from a healthy donor recruited by the Oxford Parkinson’s Disease Centre [55] and cultured with feeder-free TeSR-E8 media (STEMCELL) on tissue culture plates coated with Matrigel (Corning). Macrophages (iPSMs) were differentiated as described before [10]. Briefly, iPSCs were transferred into AggreWell plate (STEMCELL) with E8 media (Gibico) supplemented with 50 ng/mL BMP4 (ThermoFisher), 50 ng/mL VEGF (ThermoFisher) and 20 ng/mL SCF (Miltenyi Biotech) for 4 days to generate Embryoid Bodies (EBs) which were then used for generation of macrophage precursor in XVIVO-15 media (Lonza) supplemented with 25 ng/mL IL-3 (Gibco), GlutaMax, 2-mercaptoethanol and 100 ng/mL M-CSF.

### ATAC-seq

Omni-ATAC-seq was performed as described [56]. Cells (~ 50,000) were scraped from a well of a 6-well plate and then spun down at 400 g for 10 min at 4 °C. Cells were washed with 1x PBS buffer, lysed in 50 μl of lysis buffer (10 mM Tris-HCL pH 7.4, 10 mM NaCl, 3 mM MgCl_2_, 0.01% Digitonin, 0.1% Tween-20 and 0.1% NP40) for 3 min on ice, added with 1 ml Wash buffer (10 mM Tris-HCL pH 7.4, 10 mM NaCl, 3 mM MgCl_2_ and 0.1% Tween-20) and spun down at 500 g for 10 min at 4 °C. The cell pellet were resuspended in the Transposition Mixture (22.5 μl TD buffer, 2.5 μl Tn5 Transposase, 16.5 μl PBS, 0.5 μl % Digitonin, 0.5 μl 10% Tween-20, 5 μl NF-free H2O) and incubated for 30 min at 37 °C. The reaction was stopped by adding 250 μl of DNA Binding Buffer (Qiagen MinElute Kit), and the DNAs were purified by MinElute Reaction Cleanup Kit (Qiagen) and eluted in 23 μL elution buffer. To determine the appropriate cycle number for library amplification, qPCR was carried out using 2 μL of purified DNA with 1 μL each Nextera primers Ad1_noMX/Ad2.1 (25 μM), 10 μL 2X HiFi PCR Master Mix (NEB), 0.2 μL 100X SYBR Green, and 5.8 μL H2O. The libraries were purified using a Min-Elute PCR purification kit (Qiagen). Purified DNAs were quantified by Qubit assays (ThermoFisher) and quality-controlled using an Agilent TapeStation. Libraries were amplified for optimized cycles and were subjected to sequencing using a HiSeq4000 platform (Illumina).

### ChIP-seq for histone modifications

ChIPm was carried out on formaldehyde fixed MDMs from healthy donors following the ChIPmentation protocol described by [57]. Briefly, cells (~ 120,000) were cross-linked by 1% formaldehyde for 10 min, followed by 5 min quenching in 0.25 M glycine. Cells were lysed using SDS Lysis Buffer (0.25% SDS, 1 mM EDTA, 10 mM Tris.HCl pH 8 and 1x Protease Inhibitor), and sonicated using a Covaris sonicator (ChIP-5%DF-8 min program). Chromatin was re-suspended in ChIP Equilibration Buffer (1.66% TritonX100, 1 mM EDTA, 10 mM Tris.HCl pH 8, 233 mM NaCl and 1x Protease Inhibitor) and ChIP Buffer (0.1% SDS, 1% TritonX100, 1 mM EDTA, 10 mM Tris.HCl pH 8, 140 mM NaCl and 1x Protease Inhibitor) and incubated with antibodies overnight at 4 °C on an end-over rotor. Chromatin immunoprecipitation was carried out with the following Diagenode antibodies, 2 μg of H3K27ac (Cat# C15410196, RRID:AB_2637079) and 1 μg of H3K4me3 (Cat# pAb-003-050, RRID:AB_2616052). The complex co-precipitates were captured by Protein A/G magnetic beads for 2 h at 4 °C with rotation, followed by salt washes and tagmentation (20 μl TD buffer, 1 μl Tn5 Transposase and 9 μl NF-free H2O). Chromatin and the input control were eluted and reverse cross-linked. DNAs were purified, quantified and sequenced as describe above.

### RNA extraction, qRT-PCR and RNA-seq

Cells were lysed and total RNA was prepared using the Monarch Total RNA Miniprep Kit (NEB #T2010) according to the manufacturer’s protocol. cDNA was synthesized using LunaScrip RT SuperMix Kit (NEB# E3010). Quantitative real-time reverse transcription PCR (qRT-PCR) was carried out with SYBR Green Real-Time PCR Master Mix (Qiagen) in a CFX-96 C1000 platform (Rio-Rad). The transcript levels were normalized by the readings for GAPDH (see Additional file 2: Table S8 for primer sequences). RNA-seq library was prepared using a standardized rRNA depletion and dUTP protocol (NEB) followed by sequencing on either a HiSeq4000 or NextSeq500 platform (Illumina) at the Oxford Genomics Centre (Wellcome Centre for Human Genetics, Oxford, UK).

### RNA-Seq data analysis

RNA Sequencing reads were trimmed using Trim Galore (version 0.6.2), and mapped to human genome assembly hg38 using the HISAT2 (version 2.1.0). The aligned Binary-sequence Alignment Format (BAM) files were used to determine the transcript counts through featureCounts (version 1.6.2) and GENCODE annotations (release 31). The bigwig files normalized by RPKM (Reads Per Kilobase per Million mapped reads) were generated using the bamCoverage function of deepTools (version 3.3.1). For gene differential expression analysis, the raw read counts were used as input into the R package DESeq2 (version 1.28.1) for pair-wise comparisons. The exonic regions with read counts ≥10 in at least 30% of samples were used as input into the R package DEXSeq (version 1.32.0). Genes with fold change > 2 and FDR < 0.05 as per condition were considered as differentially expressed.

### Genome-wide epigenetic profiling

Sequencing reads for chromatin accessibility (ATAC) and histone modifications (H3K27ac and H3K4me3) were aligned to human genome assembly hg38 using bowtie2 (version 2.2.5). The resulting BAM files were filtered to remove non-uniquely mapped reads, non-properly paired reads, reads mapped to mitochondrial chromosome, duplicate reads and reads with a mapping quality score less than 30 using Picard (version 2.0.1) and Samtools (version 1.9). Peaks were called using MACS2 (version 2.1.0) [58] with the appropriate input dataset (paired non-ChIP data were used as controls for histone modifications). ATAC peaks were called using parameters --nomodel --shift -100 --extsize 200, and H3K27ac and H3K4me3 peaks were called using --bw 200. The normalized bigwig files showing the average sequencing depth across replicates were generated using wiggletools and wigToBigWig. Peaks that overlay the ENCODE Blacklist and with *p* value >1e−08 were filtered out. Peaks called in at least 30% of samples were defined as recurrent and merged as a list of coordinates to count the number of overlapping reads in each condition using htseq-count (version 0.6.1). Finally, the reads were normalized using DESeq2, and then pair-wise comparisons were performed to determine the differential (fold change > 2, FDR < 0.05) ATACs and histone modifications per condition. Potential batch effect or technical variable was examined by principal component analysis and was included as a covariate in DESeq2 design formula.

### TF motif analysis

Enrichment of known TF motifs within the differential ATAC peaks was calculated using the HOMER [47] (version 4.10) findMotifsGenome.pl command with default parameters. All recurrent ATAC peaks were used as the background. HOMER annotatePeaks.pl with default parameters was used to search each peak for a given de novo motif.

### eQTLs, GWAS traits and summary statistics

eQTL summary data were downloaded from eQTL Catalogue (https://www.ebi.ac.uk/eqtl/) [59]. The significant eQTLs with an association *p* value ≤1e−05 were considered for the analysis. GWAS lead SNPs were downloaded from GWAS Catalog [60] on August 5, 2020. The SNPs with an association *p* value ≤5e−08 were considered for the downstream analysis. We retrieved the associated proxy SNPs (*r*^2^ ≥ 0.8) for the lead SNPs in Europeans within a 500-kb window using the 1000 Genomes phase 3 data through PLINK 1.9. We used the GWAS Catalog Ontologies for mapping of reported traits to Parental and Experimental Factor Ontology (EFO) terms. The enrichment analysis was performed using hypergeometric test (PHYPER function as implemented in R) and multiple hypotheses testing by FDR correction. We used the stratified LD Score Regression [24] to estimate partition heritability based on pre-defined genome-wide annotation sets and the context-specific macrophage annotations and determined the enrichment of an annotation to be the proportion of SNP heritability (${\mathrm{h}}_{\mathrm{g}}^2$) divided by the proportion of SNPs in this annotation. Briefly, we obtained the baseline model LD scores, the SNP reference panels (1000 Genomes Phase 3 in Europeans) and the GWAS summary statistics from https://alkesgroup.broadinstitute.org/LDSCORE/. We converted the macrophage differential and non-differential enhancer regions to hg19 coordinates using UCSC tool liftOver. We generated the functional annotation files and computed the LD scores using make_annot.py as described in https://github.com/bulik/ldsc.

### eRNA analysis

eRNA analysis was performed on ± 1000 bp regions centred on the distal ATAC-seq peaks. Specifically, the midpoint of each ATAC peak was extended with 1000 bp from left and right. The regions that overlap with the gene boundaries (± 3000 bp from both transcription start site and end site) of the MDMs expressed genes (maximum TPM ≥1 across samples; *n* = 19,439) were filtered out. The remaining ATAC peaks (distal; *n* = 24,812) were used as coordinates to count the number of uniquely mapped total RNA-seq reads in each condition via the multicov function of bedtools (version 2.27.0). Those regions with RNA expression level of CPM (counts per million mapped reads) ≥ 1 were defined as eRNAs. The raw read counts for each eRNA across treatment conditions were used as input into DESeq2 for pair-wise comparisons. The strandedness of the RNA-seq reads was extracted using Samtools, and the normalized bidirectional bedgrapgh files were generated using Bedtools and Bedops and visualized through the WashU or UCSC Epigenome Browser.

### Lentivirus production

Lentiviral particles were generated by cotransfecting HEK293-FT cells (maintained in DMEM media with 10% FBS) with virus packaging vectors psPAX2 (Addgene #12260), pCMV-VSV-G (Addgene #8454) and the vector expressing sgRNA (Addgene #62988) in equimolar ratios, as described previously [10]. Transfection was performed using jetPRIME reagent (Polyplus). Virus supernatant was collected in 48 h and 72 h post-transfection, filtered with a 0.45-μm membrane filter (Millipore), ultracentrifuged at 29,000 rpm for 2 h at 4 °C. The pellet was resuspended in PBS with 1.5% BSA, aliquoted and stored at − 80 °C. Lentivirus were titered by determining the BFP-positive cells after transduction through flow cytometric analysis. We calculated the infectious units (IU) per μl, and used the viral volume that results in 50–60% transduction efficiency.

### CRISPRi-mediated enhancer silencing

We designed sgRNAs within the differential ATAC peaks and selected top ranked gRNAs based on on-target and off-target scoring metrics through FlashFry [61]. For the human U6 promoter-based transcription, a guanine (G) base was added to the 5′ of the sgRNA when the 20 bp guide sequence did not begin with G. The oligo sequences for the sgRNA synthesis are listed in Additional file 2: Table S8. A KOLF-C2 iPSCs line expressing dCas9-KRAB under the control of a CAG promoter, targeted to the AAVS1 locus, was generated and differentiated into iPSMs as described above. One million precursor macrophages were transduced with sgRNA-containing lentivirus in the presence of polybrene (4 μg/mL) and VPX-VLPs by spinfection at 800 g for 2 h at 37 °C. Transduced cells were maintained in XVIVO-15 media (Lonza) supplemented with 100 ng/mL M-CSF and assayed in 6 or 7 days.

## Supplementary Information

**Additional file 1: Fig. S1.** Context specific macrophage epigenetic states. **Fig. S2.** Enrichment of context-specific eQTLs within differential ATAC peaks. **Fig. S3.** GWAS enrichment and macrophage chromatin state. **Fig. S4.** Drug target prioritization (priority index, Pi) for immune disease risk genes in macrophages. **Fig. S5.** Differential gene expression and exon usage in MDMs and iPSMs. **Fig. S6.** Differential chromatin accessibility in MDMs and iPSMs. **Fig. S7.** CRISPR interference-based enhancer inactivation for *KLF4* in iPSMs. **Fig. S8.** Silencing a differential enhancer in the *SLAMF1/CD48* locus reduces LPS-mediated induction for SLAMF1 protein. **Fig. S9.** Targeting the non-differential enhancers in the *SLAMF1/CD48* and *IL2RA/RBM17* loci.

**Additional file 2: Table S1.** Context-specific macrophage epigenetic states and eQTLs (related to Figs. 1 and 2). **Table S2.** Enhancer RNAs identified in MDMs (related to Fig. 3). **Table S3.** Enrichment of functional annotations in GWAS signals using stratified LD score regression (related to Fig. 4). **Table S4.** Immune GWAS risk SNPs within differential macrophage enhancers linking differentially expressed genes (related to Fig. 4). **Table S5.** Pi ratings for immune disease risk genes in macrophages (related to Fig. 5); Table S6: 164,381 ATAC consensus peaks in CD4/CD8/CD14/CD19/MDMs/iPSMs cells (related to Fig. 6). **Table S7.** Differential MDM ATACs upon HD vs. UT and/or LDHD vs. HD, and their status in iPSMs (related to Fig. S6). **Table S8.** Oligos sequences used in this study (related to Fig. 7, S7 and S9).

**Additional file 3.** Peer review history.

## Data Availability

The sequencing datasets generated and analysed during the current study are available in the Gene Expression Omnibus (GEO) repository (GSE172116) [62].
