# Supplementary material for: Epigenomic analysis reveals a dynamic and context-specific macrophage enhancer landscape associated with innate immune activation and tolerance
Source: Genome Biol. 2022 Jun 24;23:136. doi: 10.1186/s13059-022-02702-1 (PMC9229144; doi:10.1186/s13059-022-02702-1)
Supplement: Supplementary file 1 — Additional file 1: Fig. S1. Context specific macrophage epigenetic states. Fig. S2. Enrichment of context-specific eQTLs within differential ATAC peaks. Fig. S3. GWAS enrichment and macrophage chromatin state. Fig. S4. Drug target prioritization (priority index, Pi) for immune disease risk genes in macrophages. Fig. S5. Differential gene expression and exon usage in MDMs and iPSMs. Fig. S6. Differential chromatin accessibility in MDMs and iPSMs. Fig. S7. CRISPR interference-based enhancer inactivation for KLF4 in iPSMs. Fig. S8. Silencing a differential enhancer in the SLAMF1/CD48 locus reduces LPS-mediated induction for SLAMF1 protein. Fig. S9. Targeting the non-differential enhancers in the SLAMF1/CD48 and IL2RA/RBM17 loci. [file 13059_2022_2702_MOESM1_ESM.docx]

**Epigenomic analysis reveals a dynamic and context-specific macrophage enhancer landscape associated with innate immune activation and tolerance**

Ping Zhang^1,2*^, Harindra E. Amarasinghe^2^, Justin P. Whalley^2^, Chwen Tay^2^, Hai Fang^2,3^, Gabriele Migliorini^2^, Andrew C. Brown^2^, Alice Allcock^2^, Giuseppe Scozzafava^2^, Phalguni Rath^2^‎, Benjamin Davies^2^ and Julian C. Knight^1,2*^

^1^Chinese Academy of Medical Science Oxford Institute, University of Oxford, Oxford, UK

^2^Wellcome Centre for Human Genetics, University of Oxford, Oxford, UK

^3^Ruijin Hospital, Shanghai Jiao Tong University School of Medicine, Shanghai, China

*to whom correspondence should be addressed (julian.knight@well.ox.ac.uk or ping.zhang@well.ox.ac.uk)

Additional file 1: Fig. S1-S9

|  |
| --- |
| **Fig. S1. Context specific macrophage epigenetic states.** (a) Pie charts show the fractions of MDMs recurrent peaks (upper) and differential peaks (lower) of epigenetics markers (left: ATAC; middle: H3K27ac; right: H3K4me3) in indicated genomic regions. (b) Bar plots showing the number of overlapped differential ATAC peaks (left panel) and H3K27ac peaks (right panel) upon LPS treatments. |

|  |
| --- |
| **Fig. S2. Enrichment of context-specific eQTLs within differential ATAC peaks.** (a) Bar plot showing the percentages of all common SNPs, MDMs eQTLs, iPSMs eQTLs and sepsis eQTLs located in recurrent ATAC peaks (present in >30% samples). p-values are depicted and were calculated using a two-tailed Fisher test. (b) Forest plot showing the enrichment of context-specific eQTLs within differential ATAC peaks compared with eQTLs that were only identified in naïve state. The odds ratio and p-values were calculated using a two-tailed Fisher test. To ensure the eQTLs in LD were counted only once within an ATAC region, we selected the most significant eQTL for each ATAC from a given eQTLs dataset. *** p < 0.001; **p<0.01; *p<0.05; n.s: not significant. |

|  |
| --- |
| **Fig. S3. GWAS enrichment and macrophage chromatin state.** (a) Enrichment of GWAS lead SNPs that are located in differential ATACs amongst each trait relative to other traits (n=750; traits that have lead SNPs in at least 10 differential ATACs). The horizontal green dash line represents the Bonferroni-adjusted p value of 0.05. (b) Bar plot showing the percentage of GWAS lead SNPs that have overlap with differential ATAC peaks in each enriched trait (top10 in a). Numbers of lead SNPs (number of SNPs in differential ATAC peaks/total number of SNPs) in each trait are indicated next to the bars. (c-d) Example of differentially expressed genes (*TNFSF15*, *STAT3*, *HLA-DRA*, *IL12A, IRF1* and *LINC00243*), which have nearby differential ATAC peaks harbouring GWAS lead SNPs (highlighted in purple bars). The average sequencing depth (normalized per million mapped reads) for RNA (orange), ATAC (purple), H3K27ac (brown) and H3K4me3 (green) in differing treatment conditions (UT, HD or LDHD) are shown on the y axis. |

|  |
| --- |
| **Fig. S4. Drug target prioritization (priority index, Pi) for immune disease risk genes in macrophages.** Violin plot showing the distribution of the Pi rating scores of risk genes linking differential macrophage ATACs (blue), and all the other genes that were computed by Pi rating (grey) across 14 immune traits. The median Pi rating scores in each gene group are highlighted with horizontal grey lines. |

|  |
| --- |
| **Fig. S5. Differential gene expression and exon usage in MDMs and iPSMs.** (a) Venn diagram showing the overlap of differentially expressed genes (DE genes) upon LPS treatment (FDR < 0.05; fold change > 2) between iPSMs and MDMs. (b) Pathway enrichment analysis using the common DE genes versus all analysed genes through ConsensusPathDB-human interaction database. The top 10 enriched pathways and gene names of part of enriched genes (within each bar) in each pathway are shown. (c) Chart showing a correlation matrix of 1,618 common DE genes in iPSMs and MDMs between different LPS responses. The correlation and significance levels were calculated and plotted by R GGally package. (d) Venn diagram showing the overlaps of differentially exon usage (DEUs) upon LPS treatment (FDR < 0.05; fold change > 2) between iPSMs and MDMs. 221 common LPS-induced DEU events in 110 genes were identified. (e) Chart showing a correlation matrix of 221 common differentially exons in iPSMs and MDMs between different LPS responses. (f) Bar plot showing the number of common differential exons (y-axis) and the directions of fold change upon LPS response and tolerance. For example, “-+-+” indicates an exon is downregulated upon LPS response and upregulated upon LPS tolerance in both iPSMs and MDMs. (g-h) Example of differential exon usages in immune-related genes *NCOA7* (upper left), *RASSF5* (upper right), *NFATC1* (lower left) and *BCL6* (lower right). The relevant DEUs for each gene are highlighted in grey. |

|  |
| --- |
| **Fig. S6. Differential chromatin accessibility in MDMs and iPSMs.** (a) PCA plot showing the chromatin accessibility in iPSMs (red) and MDMs (Cyan). ATAC-seq reads were counted by using the 70100 recurrent peaks identified in MDMs. The top 10% highly variable ATAC regions (ntop=7010) were selected for the PCA analysis using plotPCA function from DESeq2. (b) PCA plot showing the chromatin accessibility in iPSMs (triangle) and MDMs (asterisk) across the treatment conditions. The batch variation from differing cell type and donors were removed by using the removeBatchEffect function from limma. (c-d). Scatter plots showing log2 fold change upon LPS response (c) or tolerance (d) between MDMs (x-axis) and iPSMs (y-axis). Only the differential ATAC peaks identified in MDMs (Fold change >2 and FDR < 0.05) are shown. The significant ATACs in iPSMs (FDR < 0.05) that also showed the same change directions as MDMs are coloured in red (upregulated) or blue (downregulated). See also **Additional file 2: Table S7**. |

|  |
| --- |
| **Fig. S7. CRISPR interference-based enhancer inactivation for *KLF4* in iPSMs.** (a) Workflow for dCas9-KRAB mediated silencing of non-coding regulatory elements (see Methods). (b) *KLF4* locus showing a differential enhancer (highlighted in a green bar) associated with LPS response and tolerance in both MDMs and iPSMs. The sgRNA site for CRISPRi is highlighted by a red arrow. (c) The surrounding genes (±2Mbp) of the *KLF4* enhancer. The average sequencing depth for RNA-seq of MDMs from 6 donors are shown on the y axis. (d) Differences in expression levels of genes surrounding the *KLF4* enhancer (±2Mbp) upon LPS response (upper panel) and tolerance (lower panel). Gene expressions were measured by RNA-seq and the differentially expressed genes in each condition (FDR < 0.05) are highlighted in red. (e) Bar plot of *KLF4* expression, measured using qRT-PCR normalised to *GAPDH* (2^–∆∆Ct^) in CRISPRi edited iPSMs with either a non-target sgRNA control (green bars) or sgRNAs targeting the distal *KLF4* enhancer (red bars). Error bars represent SEM of 4 independent replicates. *P* value was calculated by two-tailed Student's t-test. |

|  |
| --- |
| **Fig. S8. Silencing a differential enhancer in the *SLAMF1/CD48* locus reduces LPS-mediated induction for SLAMF1 protein.** (a-b) Flow cytometric histograms showing the expression of SLAMF1 (also called CD150; upper panel) and CD48 (lower panel) in CRISPRi-edited iPSMs (a: non-target control sgRNA; b: SLAMF1-enahncer sgRNA) upon stimulations with HD (cyan) or LDHD (red). Cells were stained with CD150-PE and CD48-APC antibodies before and after fixation/permeabilisation to capture both the cell surface and intracellular expression. Briefly, cells were harvested using 5 mM EDTA/PBS followed by live/dead cell staining (Fixable Green Dead Cell Stain Kit; ThermoFisher), and resupsended in antibody mix (PE anti-human CD150 and APC anti-human CD48, BioLegend; or their corresponding isotype controls) for cell surface staining. Cells were then fixed and permeabilised (Fixation/Permeablization Kit, BD Biosciences) for staining the intracellular proteins using the same antibody mix as mentioned above. Finally cells were resuspended in BD Perm/Wash buffer and analysed for CD150/CD48 expression using the BD X-20 Flow Cytometry Analyser. (c) Quantifications of the CD150 or CD48 positive cells across the treatment conditions. Percentages of CD48 (purple) or CD150 (orange) positive cells were normalised to the naïve UT state of cells with either non-targeted control sgRNA or with the SLAMF1-enhancer sgRNA. |

|  |
| --- |
| **Fig. S9. Targeting the non-differential enhancers in the *SLAMF1/CD48* and *IL2RA/RBM17* loci** (a, c) Targeting the non-differential enhancers (blue) and the differential ehancers (grey) in the SLAMF1/CD48 (a) and IL2RA/RBM17 (c) loci using CRISPRi in iPSMs. See also Fig.7a. (b, d) Bar plots of fold change, measured using qRT-PCR normalised to *GAPDH* (2^–∆∆Ct^) and a non-target sgRNA control in iPSMs upon UT (left panels) or HD (right panels) (see **Additional file 2: Table S 8** for sgRNA sequences). Error bars represent SEM of 3 independent replicates. *P* value was calculated by two-tailed one-sample t-test. n.s: not significant. |
