# Supplementary material for: Epigenomic analysis reveals a dynamic and context-specific macrophage enhancer landscape associated with innate immune activation and tolerance
Source: Genome Biol. 2022 Jun 24;23:136. doi: 10.1186/s13059-022-02702-1 (PMC9229144; doi:10.1186/s13059-022-02702-1)
Supplement: Supplementary file 3 — Additional file 3. Peer review history. [file 13059_2022_2702_MOESM3_ESM.docx]

**Review history**

**First round of review**

**Reviewer 1**

The authors studied the epigenetic profiles of macrophages upon primary (high dose) vs secondary (low dose then high dose) stimulation with LPS, which represent acute response and tolerance, respectively. They performed RNA-seq, ATAC-seq and ChIP-seq for commonly studied histone modifications. They also quantified eRNA expression from total RNA-seq near ATAC-seq peaks that are distal to genes. They further validated the gene regulatory potential of ATAC-seq peaks that were differential and fit certain patterns using CRISPRi. They also have a section about the similarities between monocyte-derived macrophages from primary blood (MDMs) and iPSC derived macrophages (iPSMs) suggesting the utility of iPSMs in perturbation experiments such the CRISPRi they performed.

- A more appropriate way to do eQTL enrichment analysis (Fig 2 and S2) while accounting for LD is to employ LD Score Regression (LDSC) and its extensions. The same goes for GWAS analysis. For GWAS analysis it may also make sense to report the percentage of independent GWAS signals/loci that have overlap with differential ATAC peaks.
- In order to putatively link distal and/or differential ATAC regions to genes, the authors could have easily made use of existing looping information such as promoter capture Hi-C and HiChIP in macrophages and monocytes. This may also provide more credibility to the specificity of distal eQTL associations, for example, for KLF4 and for eRNA to gene mapping in Fig3e.
- A systematic comparison of the epigenetic profiles of MDMs vs iPSMs is missing. Showing that B and T cells cluster away from macrophages is not that informative, a detailed comparison between MDM and iPSM samples is needed. DEG analysis shows quite a substantial number between iPSM and MDM upon LPS treatment.
- For Figure 7a and 7d, an important control would have been to target other enhancers in the same locus that do not have differential ATAC peaks and/or eRNA to show whether the reduction in expression is specific to the readily targeted enhancers. Either way the results go, this would be an informative experiment.


Minor
- Line 93: majority of these accessible ATACs -> a substantial fraction
- What is the source of the "47 TFs with known sequence recognition motifs"? There certainly are more such TFs but did they subset them to ones expressed in macrophages or relevant somehow? This has to be explained.
- For completeness of this manuscript, the authors should briefly describe what "Pi" does.
- Line 218: The argument that KLF4 is the only DEG conflicts with the figure itself where there is a zinc finger gene that is also DE.
- The discussion of Fig7 jumping from one locus to the other then to CRISPR validation of each is a bit confusing. Would have been easier to follow if discussion of all data on one locus is done and moved onto the second one.
- I am not able to understand the point in discussion of exon usage in general.
- Fig. 3: Distal ATAC and distal enhancer region definitions are not clear. What is the distance threshold from corresponding TSS?

**Reviewer 2**

Zhang et al. characterize the innate immune epigenetic and transcriptional landscape of human macrophages. They use an acute model of LPS and a low LPS dose with a subsequent high LPS dose to study the molecular changes in a tolerant state. Using a combination of RNAseq, ATACseq, and determination of epigenetic marks for enhancer and promoter activity, the identify regulatory enhancers, and demonstrate that distinct epigenetic changes enable the stratification of the relationships between transcription factor binding, enhancer accessibility, gene expression, and exon usage. Further they connect this usage with disease-associated genetic variants.

In summary, this is a very comprehensive study giving insights into the molecular changes involved in macrophage tolerance. This will serve as a great resource for the macrophage community. Yet, few points need to be addressed:

1. Using iPSC-derived macrophages is a big strength of the manuscript that needs to be addressed in more detail. MDMs and iPSMs represent two distinct arms of macrophage development, thus representing also distinct cell types with specialized functions (e.g. see review PMID: 33542457). iPSMs go through the EB stage, which represents the yolk sac stage of fetal-derived macrophages. In contrast, MDMs stem from hematopoietic stem cells. These macrophage subpopulations exist side by side in every tissue and their function in disease onset and progression may not be the same. In the current version, the authors try to highlight the similarities between MDMs and iPSMs, however when looking at the data in more detail, these cells are quite distinct from each other on the transcriptional and epigenetic level. This fact should be further analysed and highlighted. Although both MDMs and iPSMs share a certain molecular program (as expected), the authors should highlight that ontogeny of macrophages actually drives a distinct transcriptional and epigenetic program.
2. The approach of CRISPRi is intriguing. However, macrophages typically to not express hematopoietic stem cell markers such as CD150 and CD48 in non-diseased conditions. The authors should provide some validation on protein level (e.g. via flow cytometry) for all 3 conditions.

Minor points:
a. Figure 3f: consider splitting the different conditions for ATAC, H3K27ac, and H3K4me3. It is very difficult to see changes between the black, red and cyan colors
b. The legend in 3f has a typo. It should be IL10RA and not IL20RA

**Authors’ response to reviewers**

***Reviewer reports:***

***Reviewer #1:*** *The authors studied the epigenetic profiles of macrophages upon primary (high dose) vs secondary (low dose then high dose) stimulation with LPS, which represent acute response and tolerance, respectively. They performed RNA-seq, ATAC-seq and ChIP- seq for commonly studied histone modifications. They also quantified eRNA expression from total RNA-seq near ATAC-seq peaks that are distal to genes. They further validated the gene regulatory potential of ATAC-seq peaks that were differential and fit certain patterns using CRISPRi. They also have a section about the similarities between monocyte-derived macrophages from primary blood (MDMs) and iPSC derived macrophages (iPSMs) suggesting the utility of iPSMs in perturbation experiments such the CRISPRi they performed.*

We thank Reviewer 1 for these comments and the subsequent helpful suggestions, which have been very helpful in improving the manuscript.

*- A more appropriate way to do eQTL enrichment analysis (Fig 2 and S2) while accounting for LD is to employ LD Score Regression (LDSC) and its extensions. The same goes for GWAS analysis. For GWAS analysis it may also make sense to report the percentage of independent GWAS signals/loci that have overlap with differential ATAC peaks.*

As suggested by the reviewer, we used the stratified LD Score Regression to estimate partition heritability based on pre-defined baseline annotation sets and the context-specific macrophage enhancers, and determined the enrichment of an annotation in GWAS signals to be the proportion of SNP heritability (h!) divided by the proportion of SNPs in this

!

annotation as described in [1] (see Methods section on page 15). As shown in the new **Fig.4b**, we observed significant enrichments of differential ATAC regions in GWAS signals for Crohn’s disease, ulcerative colitis, inflammatory bowel disease traits and multiple sclerosis (>70X on average), but not for the non-differential ATAC regions. Consistent with the results obtained by directly comparing differential ATACs with non-differential ATACs in **Fig.4a**, these significant enrichments occur in immune-relevant traits but not in other diseases and quantitative traits such as body mass index and height measurements (see **Fig. 4a-b** and the Results section on pages 6 and **Supplementary Table** S3).

We also agree with the reviewer that it is important to report the percentage of independent GWAS loci that have overlap with differential ATAC peaks. We therefore restricted our analysis by using the GWAS lead SNPs and report the number and percentage of GWAS lead SNPs for the top10 enriched disease/traits (see the new **Fig.S3a-b** and Results section on page 6). On average, about 4% of the independent risk loci have overlap with differential ATAC peaks across the top enriched disease traits (**Fig.S3a-b**). To ensure the eQTLs in LD were counted only once within an ATAC region, we now have selected the most significant eQTL within each ATAC from a given eQTLs dataset for the analysis in **Fig. S2.** Similarly, in the new **Fig. 2d**, we attributed the most significant eQTL-gene pair for each ATAC peak before determining the enrichment of DE genes linking differential ATACs versus non- differential ATACs. We have added this information in the Results section on page 5 and Figure legends for **Fig. 2d and S3a** of the revised manuscript.

*- In order to putatively link distal and/or differential ATAC regions to genes, the authors could have easily made use of existing looping information such as promoter capture Hi-C and HiChIP in macrophages and monocytes. This may also provide more credibility to the specificity of distal eQTL associations, for example, for KLF4 and for eRNA to gene mapping in Fig3e.*

We thank Reviewer 1 for the helpful comments and we agree that the chromatin looping information could provide more credibility to our prioritised gene-enhancer associations. Many of these enhancers and eRNAs are highly dynamic and context-specific - for example the KLF4 enhancer that is active upon acute LPS stimulation and tolerised upon LPS re- exposure – such that we would expect the looping interactions between the enhancer and target gene promoters in many instances only to occur upon acute stimulation but not in naïve or tolerised condition. However there is no available PCHiC or HiChIP dataset in relevant cells (primary CD14 monocytes, MDMs or iPSMs) upon acute LPS stimulation.

Studies have showed the prolonged immune stimulation, e.g, LPS for 24 hours [2], triggers tolerance-like phenotypes. Indeed, we observed the strong KLF4 eQTL association identified in monocytes upon LPS for 2 hours was completely lost upon 24 hours LPS (red box of the Figure below). We have explored this with available PCHiC datasets [3] in monocytes/macrophages of native state or with prolonged LPS treatment (>18h) but did not observe a significant loop that interacts with the KLF4 enhancer (see the Figure below), which likely reflects that informative PCHiC datasets for acute (2h) LPS stimulation are not yet available.

**Figure:** The putative promoter-enhancer looping interactions within the KLF4 locus. The cell types and treatment conditions of PCHiC (promoter capture Hi-C) data are shown on the y axis. We obtained the publicly available PCHiC data in relevant cell types (primary CD14 monocytes and macrophages) from [3]. We utilised the putative causal interactions (CHiCAGO score ≥ 5; confidence score for interactions as described in [3, 4]) and lifted over the coordinates to hg38 using liftOverBedpe (https://github.com/dphansti/liftOverBedpe) and visualized the bedpe file (longrange formart) within the enhancer region (highlighted in grey bar).

- *A systematic comparison of the epigenetic profiles of MDMs vs iPSMs is missing. Showing that B and T cells cluster away from macrophages is not that informative, a detailed*

*comparison between MDM and iPSM samples is needed. DEG analysis shows quite a substantial number between iPSM and MDM upon LPS treatment.*

We thank Reviewer 1 for the helpful comments. We have now performed a systematic comparison of epigenetic profiles between MDMs and iPSMs upon both the acute LPS response and tolerance conditions. To directly compare the two models, we first counted the ATAC-seq reads for iPSMs by using the recurrent peak regions identified in MDMs (n=70100). We observed the first principal component clearly separated MDMs from iPSMs (**Fig. S6a)**, which is consistent with previous studies for transcriptome profiles [5-7]. When we corrected variance due to the differing cell types and donors, samples from the two cellular models tend to be clustered together across the treatment conditions (**Fig. S6b**). The second principal component however still slightly separated native and LPS-treated samples between iPSMs and MDMs (black arrows; **Fig. S6b**). In total, we determined 56.7% (2898 out of 5019 upon LPS response) and 25.4% (331 out of 1305 upon LPS tolerance) of the differential ATACs identified in MDMs were also differential in the same direction in iPSMs (**Fig. S6c-d**; **Supplementary Table S7**). The above new results have been included in the Results section on page 8 of the revised manuscript.

- *For Figure 7a and 7d, an important control would have been to target other enhancers in the same locus that do not have differential ATAC peaks and/or eRNA to show whether the reduction in expression is specific to the readily targeted enhancers. Either way the results go, this would be an informative experiment.*

We agree with the reviewer that it would be an important control to target the non-differential enhancers in the same locus. We therefore prepared the sgRNA lenti-viral particles against the non-differential enhancers and the differential enhancers residing in the SLAMF1/CD48 and IL2RA/RBM17 loci (as highlighted in blue and grey bars respectively in **Fig. S9a and Fig. S9c**). We titered the viruses to make sure ≥50% iPSM-KRAB cells were transduced with the sgRNA viruses. By targeting the corresponding prioritised differential enhancers, we observed clear reductions of expression for the target genes SLAMF1 and IL2RA upon LPS stimulation (**Fig. S9b and Fig. S9d;** also see **Fig. 7g-h**). In contrast, by targeting the non- differential enhancers, we did not see significant changes in expression of the flanking genes (**Fig. S9b and Fig. S9d** blue bars). We have added this result on page 9 of the revised manuscript.

*Minor*

- *Line 93: majority of these accessible ATACs -> a substantial fraction*

Thank you. We have revised it accordingly.

- *What is the source of the "47 TFs with known sequence recognition motifs"? There certainly are more such TFs but did they subset them to ones expressed in macrophages or relevant somehow? This has to be explained.*

We thank the reviewer for this feedback. We used all 428 motifs curated in HOMER (version 4.10) [8] for the analysis (as described in the Methods). In the revised manuscript, we have included the total number of TF motifs on page 4 of the Results section: “We identified 47 out of 428 known TF sequence recognition motifs were enriched in differential ATAC regions.”

- *For completeness of this manuscript, the authors should briefly describe what "Pi" does.*

We agree with the reviewer and have included the following in the revised manuscript (Results section on page 7): “We recently developed a drug target prioritization pipeline (Priority index; Pi) to identify potential therapeutic targets in immune traits using immune disease GWAS, annotated with functional immunogenomics and knowledge of network connectivity [9]*”.*

- *Line 218: The argument that KLF4 is the only DEG conflicts with the figure itself where there is a zinc finger gene that is also DE.*

Thank you. We have clarified this on page 9 of the Results: “Consistently, among all genes that lie approximately 2 Mb on either side of the enhancer, KLF4 is the only one that was significantly upregulated upon LPS response and downregulated upon tolerance (Fig. S7c- d).”

- *The discussion of Fig7 jumping from one locus to the other then to CRISPR validation of each is a bit confusing. Would have been easier to follow if discussion of all data on one locus is done and moved onto the second one.*

We thank the reviewer for the helpful comments. We have now moved the discussion of the second locus followed by its CRISPR validation on page 9 of the Results section in the revised manuscript.

- *I am not able to understand the point in discussion of exon usage in general.*

We agree and have removed this from the revised manuscript to make the Discussion section more focused.

- *Fig. 3: Distal ATAC and distal enhancer region definitions are not clear. What is the distance threshold from corresponding TSS?*

We thank the reviewer for this feedback. To distinguish eRNAs from intronic pre-mRNAs, we used the distal ATACs for eRNA calling. These distal ATAC regions were defined if they do not overlap with known expressed transcripts in MDMs (±3000 bp from both transcription start site and end site). We now have highlighted these definitions in **Fig.3a** and the Results section on page 6 of the revised manuscript.

***Reviewer #2:*** *Zhang et al. characterize the innate immune epigenetic and transcriptional landscape of human macrophages. They use an acute model of LPS and a low LPS dose with a subsequent high LPS dose to study the molecular changes in a tolerant state. Using a combination of RNAseq, ATACseq, and determination of epigenetic marks for enhancer and promoter activity, the identify regulatory enhancers, and demonstrate that distinct epigenetic changes enable the stratification of the relationships between transcription factor binding, enhancer accessibility, gene expression, and exon usage. Further they connect this usage with disease-associated genetic variants.*

*In summary, this is a very comprehensive study giving insights into the molecular changes involved in macrophage tolerance. This will serve as a great resource for the macrophage community. Yet, few points need to be addressed:*

We thank reviewer 2 for the appreciation of our study and for the helpful suggestions and comments, which clearly improved the manuscript. Please find a detailed response below.

1. *Using iPSC-derived macrophages is a big strength of the manuscript that needs to be addressed in more detail. MDMs and iPSMs represent two distinct arms of macrophage development, thus representing also distinct cell types with specialized functions (e.g. see review PMID: 33542457). iPSMs go through the EB stage, which represents the yolk sac stage of fetal-derived macrophages. In contrast, MDMs stem from hematopoietic stem cells. These macrophage subpopulations exist side by side in every tissue and their function in disease onset and progression may not be the same. In the current version, the authors try to highlight the similarities between MDMs and iPSMs, however when looking at the data in more detail, these cells are quite distinct from each other on the transcriptional and epigenetic level. This fact should be further analysed and highlighted. Although both MDMs and iPSMs share a certain molecular program (as expected), the authors should highlight that ontogeny of macrophages actually drives a distinct transcriptional and epigenetic program.*

We agree with the reviewer that the process of cellular differentiation is distinct between MDMs and iPSMs, and it is important to highlight the differences between the models. We therefore performed a systematic comparison of epigenetic profiles between MDMs and iPSMs at their native stage and stimulation conditions. Specifically, we first counted the ATAC-seq reads for iPSMs by using the recurrent peak regions identified in MDMs (n=70100). Consistent with the previous studies for transcriptome profiles [5-7], the PCA analysis showed a clear separation between iPSMs and MDMs across the samples (**Fig. S6a**). When we corrected the variance due to the differing cell types and donors, samples from the two cellular models tend to be clustered together across the treatment conditions (**Fig. S6b**). The second principal component however still slightly separated native and LPS- treated samples between iPSMs and MDMs (black arrows; **Fig. S6b**). We made a new supplementary file to report the epigenetic levels of iPMSs for all the differential ATACs identified in MDMs. In total, we determined 56.7% (2898 out of 5019 upon LPS response) and 25.4% (331 out of 1305 upon LPS tolerance) of the differential ATACs identified in MDMs were also differential in the same direction in iPSMs (**Fig. S6c-d**; **Supplementary Table S7**). These new results have been included in the Results section on page 8 of the revised manuscript.

We have also added the following in the Discussion section on page 11: “Although iPSMs and MDMs exhibited broadly conserved transcriptome and epigenetic profiles in response to LPS stimulations, we observed clear differences in numbers of DE genes, DEUs and differential enhancers between the two macrophage models, probably due to iPSMs represent primitive, tissue resident macrophages which are known to be developmentally and functionally distinct from MDMs [10, 11]. We determined that about 50% of the differential ATACs identified in MDMs showed consistent profiles in iPSMs.”

2. *The approach of CRISPRi is intriguing. However, macrophages typically to not express hematopoietic stem cell markers such as CD150 and CD48 in non-diseased conditions. The authors should provide some validation on protein level (e.g. via flow cytometry) for all 3 conditions.*

We thank Reviewer 2 for the helpful comment and suggestion. Via flow cytometry, we were able to determine the treatment responses of CD150 and CD48 and validate the enhancer activity on CD150 upon LPS stimulation on protein levels. We have included this result in the revised manuscript (on page 9 of the Results section and **Figure S8**). Indeed, we observed the expression levels of both CD150 and CD48 were relatively low in the naïve cells (isotype controls (in blue) vs. the UT sample stained with CD150/CD48 antibodies (in grey); **Fig.S8a**). Both CD150 and CD48 were induced upon LPS stimulation (HD sample (in cyan) vs. UT sample (in grey) in **Fig.S8a; Fig.S8c**), which is consistent with previous studies [12- 14]. Similar to the mRNA levels, we saw a clear tolerance phenotype for CD150 with reduced proteins upon LDHD (**Fig. S8a** upper panel in red; **Fig. S8c**) but not for CD48 with even stronger expression upon LDHD (**Fig. S8a** lower panel in red; **Fig. S8c**). When we treated the cells with sgRNAs targeting the prioritised differential enhancer as shown in **Fig. 7a**, the LPS-mediated induction of proteins was clearly reduced for CD150 (from 5.9X to 1.3X) but not for CD48 (**Fig. S8a vs. S8b; Fig. S8c**).

*Minor points:*

a. *Figure 3f: consider splitting the different conditions for ATAC, H3K27ac, and H3K4me3. It is very difficult to see changes between the black, red and cyan colors*

We agree and have split the treatment conditions for ATAC, H3K27ac and H3K4me3 into three different tracks and labeled the tracks accordingly on the y axis in the revised manuscript.

b. *The legend in 3f has a typo. It should be IL10RA and not IL20RA*

Thank you. We have corrected this typo in the revised manuscript.

# Reference

1. Finucane HK, Bulik-Sullivan B, Gusev A, Trynka G, Reshef Y, Loh PR, Anttila V, Xu H, Zang C, Farh K, et al: **Partitioning heritability by functional annotation using genome-wide association summary statistics.** *Nat Genet* 2015, **47:**1228-1235.
2. Fairfax BP, Humburg P, Makino S, Naranbhai V, Wong D, Lau E, Jostins L, Plant K, Andrews R, McGee C, Knight JC: **Innate immune activity conditions the effect of regulatory variants upon monocyte gene expression.** *Science* 2014, **343:**1246949.
3. Javierre BM, Burren OS, Wilder SP, Kreuzhuber R, Hill SM, Sewitz S, Cairns J, Wingett SW, Varnai C, Thiecke MJ, et al: **Lineage-Specific Genome Architecture Links Enhancers and Non-coding Disease Variants to Target Gene Promoters.** *Cell* 2016, **167:**1369-1384 e1319.
4. Cairns J, Freire-Pritchett P, Wingett SW, Varnai C, Dimond A, Plagnol V, Zerbino D, Schoenfelder S, Javierre BM, Osborne C, et al: **CHiCAGO: robust detection of DNA looping interactions in Capture Hi-C data.** *Genome Biol* 2016, **17:**127.
5. Alasoo K, Martinez FO, Hale C, Gordon S, Powrie F, Dougan G, **Mukhopadhyay S, Gaffney DJ: Transcriptional profiling of macrophages derived from monocytes and iPS cells identifies a conserved response to LPS and novel alternative transcription**. *Sci Rep* 2015, 5:12524.
6. Monkley S, Krishnaswamy JK, Goransson M, Clausen M, Meuller J, Thorn K, Hicks R, Delaney S, Stjernborg L: **Optimised generation of iPSC-derived macrophages and dendritic cells that are functionally and transcriptionally similar to their primary counterparts.** *PLoS One* 2020, **15:**e0243807.
7. Zhang H, Xue C, Shah R, Bermingham K, Hinkle CC, Li W, Rodrigues A, Tabita- Martinez J, Millar JS, Cuchel M, et al: **Functional analysis and transcriptomic profiling of iPSC-derived macrophages and their application in modeling Mendelian disease.** *Circ Res* 2015, **117:**17-28.
8. Heinz S, Benner C, Spann N, Bertolino E, Lin YC, Laslo P, Cheng JX, Murre C, Singh H, Glass CK: **Simple combinations of lineage-determining transcription factors prime cis-regulatory elements required for macrophage and B cell identities.** *Mol Cell* 2010, **38:**576-589.
9. Fang H, Consortium U-D, De Wolf H, Knezevic B, Burnham KL, Osgood J, Sanniti A, Lledo Lara A, Kasela S, De Cesco S, et al: **A genetics-led approach defines the drug target landscape of 30 immune-related traits.** *Nat Genet* 2019, **51:**1082- 1091.
10. Buchrieser J, James W, Moore MD: **Human Induced Pluripotent Stem Cell- Derived Macrophages Share Ontogeny with MYB-Independent Tissue-Resident Macrophages.** *Stem Cell Reports* 2017, **8:**334-345.
11. Mass E, Lachmann N: **From macrophage biology to macrophage-based cellular immunotherapies.** *Gene Ther* 2021, **28:**473-476.
12. Yurchenko M, Skjesol A, Ryan L, Richard GM, Kandasamy RK, Wang N, Terhorst C, Husebye H, Espevik T: **SLAMF1 is required for TLR4-mediated TRAM-TRIF- dependent signaling in human macrophages.** *J Cell Biol* 2018, **217:**1411-1429.
13. Moller J, Luhmann T, Chabria M, Hall H, Vogel V: **Macrophages lift off surface- bound bacteria using a filopodium-lamellipodium hook-and-shovel mechanism.** *Sci Rep* 2013, **3:**2884.
14. Barbero AM, Trotta A, Genoula M, Pino R, Estermann MA, Celano J, Fuentes F, Garcia VE, Balboa L, Barrionuevo P, Pasquinelli V: **SLAMF1 signaling induces Mycobacterium tuberculosis uptake leading to endolysosomal maturation in human macrophages.** *J Leukoc Biol* 2021, **109:**257-273.

**Second round of review**

**Reviewer 1**

The authors have addressed all my comments

**Reviewer 2**

Thank you for addressing the points raised after your first submission.
The manuscript has really improved and should be published as is.
